# Contribution by Polymorphonucleate Granulocytes to Elevated Gamma-Glutamyltransferase in Cystic Fibrosis Sputum

**DOI:** 10.1371/journal.pone.0034772

**Published:** 2012-04-04

**Authors:** Alessandro Corti, Maria Franzini, Silvana Cianchetti, Gabriella Bergamini, Evelina Lorenzini, Paola Melotti, Aldo Paolicchi, Pierluigi Paggiaro, Alfonso Pompella

**Affiliations:** 1 Dipartimento di Patologia Sperimentale, Università di Pisa, Scuola Medica, Pisa, Italy; 2 Scuola Superiore S. Anna, Pisa, Italy; 3 Fondazione Toscana G. Monasterio, CNR, Regione Toscana, Pisa, Italy; 4 Dipartimento Cardio Toracico e Vascolare, Università di Pisa, Pisa, Italy; 5 Centro Fibrosi Cistica, Azienda Ospedaliera di Verona, Verona, Italy; 6 Dipartimento di Patologia e Diagnostica, Università di Verona, Verona, Italy; University of Tübingen, Germany

## Abstract

**Background:**

Cystic fibrosis (CF) is an autosomal recessive disorder characterized by a chronic neutrophilic airways inflammation, increasing levels of oxidative stress and reduced levels of antioxidants such as glutathione (GSH). Gamma-glutamyltransferase (GGT), an enzyme induced by oxidative stress and involved in the catabolism of GSH and its derivatives, is increased in the airways of CF patients with inflammation, but the possible implications of its increase have not yet been investigated in detail.

**Principal Findings:**

The present study was aimed to evaluate the origin and the biochemical characteristics of the GGT detectable in CF sputum. We found GGT activity both in neutrophils and in the fluid, the latter significantly correlating with myeloperoxidase expression. In neutrophils, GGT was associated with intracellular granules. In the fluid, gel-filtration chromatography showed the presence of two distinct GGT fractions, the first corresponding to the human plasma b-GGT fraction, the other to the free enzyme. The same fractions were also observed in the supernatant of ionomycin and fMLP-activated neutrophils. Western blot analysis confirmed the presence of a single band of GGT immunoreactive peptide in the CF sputum samples and in isolated neutrophils.

**Conclusions:**

In conclusion, our data indicate that neutrophils are able to transport and release GGT, thus increasing GGT activity in CF sputum. The prompt release of GGT may have consequences on all GGT substrates, including major inflammatory mediators such as S-nitrosoglutathione and leukotrienes, and could participate in early modulation of inflammatory response.

## Introduction

Cystic fibrosis (CF) is an autosomal recessive disorder due to mutations in the cystic fibrosis trans-membrane conductance regulator protein (CFTR) [1], [2], a cyclic AMP-regulated anion channel primarily involved in chloride and bicarbonate transport but also permeable to other larger organic anions such as glutathione (GSH) [3], [4]. CFTR impairment has a wide impact on the functions of several tissues but, in particular, it is associated with alterations of biophysical properties of airway secretions [5] leading to chronic airway infection and inflammation, the latter mainly dominated by neutrophils [6], [7]. Such conditions are associated with increased levels of oxidative stress in the lung and several studies have therefore focused on the antioxidant/oxidants balance in CF, with particular interest on GSH and GSH-associated enzymes [1], [2]. GSH is one of the major water-soluble antioxidants and its chemical properties make it able to play a role also in mucolysis, regulation of inflammation, immune response and cell viability [1]. Interestingly, GSH concentrations are markedly reduced in CF airways and plasma [8], and several factors (e.g. chronic inflammation, oxidative stress, impaired CFTR-mediated GSH transport) may contribute to this effect.

Gamma-glutamyltransferase (GGT) is a membrane-bound enzyme involved in the metabolism and recuperation of extracellular glutathione by cells. GGT is also involved in S-nitrosoglutathione and leukotrienes metabolisms [9], [10] and several studies documented its role in promoting pro-oxidant reactions, thanks to the highly reactive GSH-derivative cysteinyl-glycine [11]. Indeed, cysteinyl-glycine can be considered as a marker of GGT activity and its ability in promoting protein S-thiolation was also shown [12].

GGT expression can be induced by oxidative stress [13], [14] and inflammatory cytokines, such as TNF-alpha, IFN-alpha and –beta (see [11] for a recent review). Interestingly, a significant increase in GGT activity was described in the bronchoalveolar lavage of young children with pulmonary inflammation due to CF [15] and such increase was interpreted as a response to inflammation-related oxidative stress, likely providing bronchial cells with a mechanism for an increased recovery of extracellular glutathione [1], [15]. Higher GGT activities were also detected *in vitro* in cultured CF cell lines [4], [16], suggesting that the GGT increase in CF lungs may be directly related with CFTR defective function. Nevertheless other non-epithelial sources should be taken into account when considering the GGT increase in CF lungs. In particular, some studies demonstrated the expression of GGT in human lymphoid cells and an increase of GGT activity was described in the granulocytic cell lineage along with cell maturation [17], during differentiation of lymphocytes [18] and monocytes/macrophages [19]. In neutrophils GGT is localized in microsomal and granular fractions and released upon neutrophils activation with calcium-ionophore A23187 [9], [17], [20].

The aim of the present work was to assess the origin and the biochemical characteristics of the GGT detectable in CF sputum in comparison with the enzyme released by activated neutrophils, in order to appraise the contribution of inflammation-derived GGT to the increased activity described in CF lungs.

## Materials and Methods

### Chemicals

Unless otherwise indicated, all reagents were from Sigma Chemical Co. (St. Louis, MO, USA).

### Ethics Statement

The study was approved by Human Ethics Committee of Azienda Ospedaliera of Verona and all subjects gave a written informed consent. A written informed consent was also obtained from the next of kin on the behalf of the minors participants involved in the study.

### Processing of cystic fibrosis sputum samples for GGT assays

Spontaneously produced sputum samples (N. 7 specimens obtained from 7 distinct CF patients) were collected from patients affected by classical cystic fibrosis attending the Cystic Fibrosis Center of Verona. The group included 4 males and 3 females with age ranging from 15 to 36 years and different severity of lung function impairment (forced expiratory volume in one second (FEV1) ranging from 29% to 86% expected value). Samples from bronchiectasis patients (2 males, 3 females; age from 61 to 78) were used as neutrophils-dominated, chronic airways inflammation control. For total GGT measurements, samples were diluted 8-fold in 10 mM Tris-HCl pH 7.8, including Triton X-100 (1% v/v) and sonicated. For soluble GGT measurements, sputum samples were diluted with an equal volume of 0.1% w/v dithiothreitol (Sputasol; Unipath, Basingstoke, UK), incubated in a shaking bath at 37°C for 15 min, then gently mixed to further dissolve mucus plugs. At the end of incubation, samples were filtered through a 53 µm nylon gauze to remove debris [21]. Filtered samples were centrifuged at 400×g (7 min, RT), then at 10,000×g (10 min, 4°C). Both soluble fraction (supernatant) and insoluble pellet (resuspended in PBS) were collected. All samples were stored at −80°C.

### Determination of low molecular weight thiols in cystic fibrosis sputum

Determination of low molecular weight thiols was performed as previously described [12] on whole sputum samples acidified with 10% trichloroacetic acid. Samples were reduced with tris(2-carboxyethyl)phosphine (Molecular Probes), and derivatized with the thiol-reagent 7-fluorobenzo-2-oxa-1,3-diazole-4-sulfonate (Fluka). Thiols concentration was determined by HPLC system.

### Cytochemical staining for GGT activity

Cystic fibrosis sputum smears were fixed in a phosphate-buffered acetone formaldehyde mixture (PBAF) and stained with gamma-glutamyl-4-methoxy-2-naphtylamide and Fast Garnet GBC as previously described [17]. Nuclei were counterstained with Mayer's hemalum solution.

### Isolation and activation of neutrophils

Neutrophils were isolated from the blood of healthy donors as described [22]. Fresh buffy coats were incubated with 1% Dextran T500. Leukocyte-rich supernatants were recovered and contaminating erythrocytes lysed with distilled water; neutrophils were separated by centrifugation on Histopaque-1077. Cell number and viability were assessed by Turk's staining and Trypan blue exclusion. All manipulations were performed under sterile conditions at 4°C. Neutrophils (5×10^6^ cells/ml) were incubated in RPMI-1640 at 37°C and challenged with 0.5 µM ionomycin (15 min) or 1 µM formyl-methionyl-leucyl-phenylalanine (fMLP, 120 min); cell viability was assessed by Trypan blue exclusion. Finally samples were centrifuged at 300×g (5 min, 4°C) then at 10,000×g (10 min, 4°C) before GGT determinations.

### Isolation of neutrophils granules on Percoll gradients

Neutrophils granules were separated as described [23]. Isolated neutrophils (2–5×10^7^ cells/ml) were pressurized in a nitrogen bomb and the samples were collected dropwise. Nuclei and intact cells were separated by centrifugation and the supernatants were stored on ice. A discontinuos Percoll gradient was prepared by stratifying three Percoll solutions with densities of 1.120, 1.090 and 1.050 g/ml. Supernatants were applied on top of the gradients and centrifuged at 37,000×g (30 min, 4°C). Four main bands were thus identified corresponding to (from bottom): α-band (containing azurophil granules), β_1_-band (specific granules), β_2_-band (gelatinase granules), and γ-band (secretory vesicles and plasma membranes). Cytosol was separated on top of upmost band. The five fractions and fractions among them were harvested through a Pasteur pipette and stored at −20°C.

### Fractional GGT analysis by high-performance gel-filtration chromatography

Determination of GGT fractions was performed as previously described [24], [25] by a FPLC system (AKTA-purified-10, GE-Healthcare). Separation and quantification of GGT fractions was performed by gel-filtration chromatography (Superose 6 10/300, GE Healthcare) followed by post-column injection of the fluorescent substrate gamma-glutamyl-7-amido-4-methylcoumarin. Intensity of the fluorescence signal was expressed in arbitrary fluorescence units (f.u.) and the area under chromatographic peaks was proportional to GGT activity.

Fractional GGT analysis on activated neutrophils supernatants and solubilised sputum samples were both performed after centrifugation at 10,000×g (30 min, 4°C) followed or not by 100,000×g (120 min, 4°C) ultracentrifugation.

### Cell lines and culture conditions

CFTR-mutated IB3-1 cells derived from bronchial epithelium of a CF patient [26] were obtained from Dr. P. Zeitlin (Johns Hopkins University, MD, USA). IB3-1 cells were routinely grown in LHC-8 medium (Gibco) supplemented with 5% (v/v) foetal bovine serum (FBS). Human alveolar basal epithelial A549 cells [27] were grown in DMEM supplemented with 10% (v/v) FBS and 2 mM L-glutamine (L-Gln). Cell lines were cultured at 37°C in a 5%/95% CO_2_/air atmosphere.

### Western blot analysis

The extracellular and cytoplasmatic levels of neutrophilic myeloperoxidase (MPO) were evaluated by western blot analysis of the solubilised sputum supernatants and cells lysates, respectively. The sputum cells were directly lysed in sample buffer (40 mM Tris-HCl pH 6.8, 183 mM β-mercaptoethanol, 1% (w/v) SDS, 5% (v/v) glycerol), heated at 95°C for 5 min and passed through a 23 gauge needle to fragment DNA. All samples were separated by 12% SDS-PAGE and gels were blotted onto nitrocellulose membrane (Hybond ECL; Amersham, UK). Membranes were stained with Ponceau S to verify loading and transfer efficiency. Nonspecific binding on the membrane was blocked with 5% (w/v) bovine serum albumin (BSA) in TBS-T buffer (0.2% Tween 20 in Tris-buffered saline pH 7.5) for 1 hour at room temperature. Membranes were incubated with 1∶1,000 dilution of rabbit polyclonal antibody raised against human MPO (Enzo Life Sciences Inc, NY, USA) or 1∶2,000 mouse monoclonal anti-GAPDH (Life Technologies, Grand Island NY, USA) in TBS-T with 1% BSA, overnight, at 4°C. Blot was washed three times in TBS-T and then incubated for 1 hour at room temperature with donkey anti-rabbit IgG secondary antibody or sheep anti-mouse IgG conjugated to horseradish peroxidase (Amersham, NJ, USA) diluted 1∶15,000 in TBS-T. Bound proteins were visualized using the ECL detection system (Amersham).

For western blot determinations of GGT, isolated neutrophils and epithelial cell monolayers – harvested in hypotonic lysis buffer (10 mM Tris–HCl, pH 7.8) – or aliquots of CF sputum were used. All samples were separated by 12% SDS-PAGE and gels were blotted onto nitrocellulose membranes. Nonspecific binding on the membrane was blocked with 5% (w/v) non-fat milk/1× PBS-0.01% Tween 20 for 30 min at room temperature. Blots were incubated overnight, 4°C, with rabbit anti-GGT IgG (1∶1000 in 2.5% (w/v) non-fat milk/1× PBS-0.01% Tween 20) directed against the C-terminal 20 amino acids of human GGT heavy chain and prepared as described [28]. Visualization of protein bands was obtained using a horseradish peroxidase-conjugated anti-rabbit IgG antibody (Santa Cruz Biotechnology, Santa Cruz, CA, USA) diluted 1∶5,000 in 2.5% (w/v) non-fat milk/1× PBS-0.01% Tween 20 (1 hour, room temperature), and the ECL detection system (Roche, Basel, Switzerland).

Bands were quantified by densitometric analysis with a Bio-Rad ChemiDoc apparatus equipped with the QuantityOne software.

### Other determinations

GGT activity was determined according to Huseby and Strömme [29]. Protein content was determined by the method of Bradford using the Bio-Rad protein assay reagent. Statistical analysis of data was performed by linear regression analyses, Student's t-test and one-way ANOVA with Newman–Keuls test for multiple comparisons.

## Results

### Characterization of GGT activity in whole CF sputum

**Figure 1:**
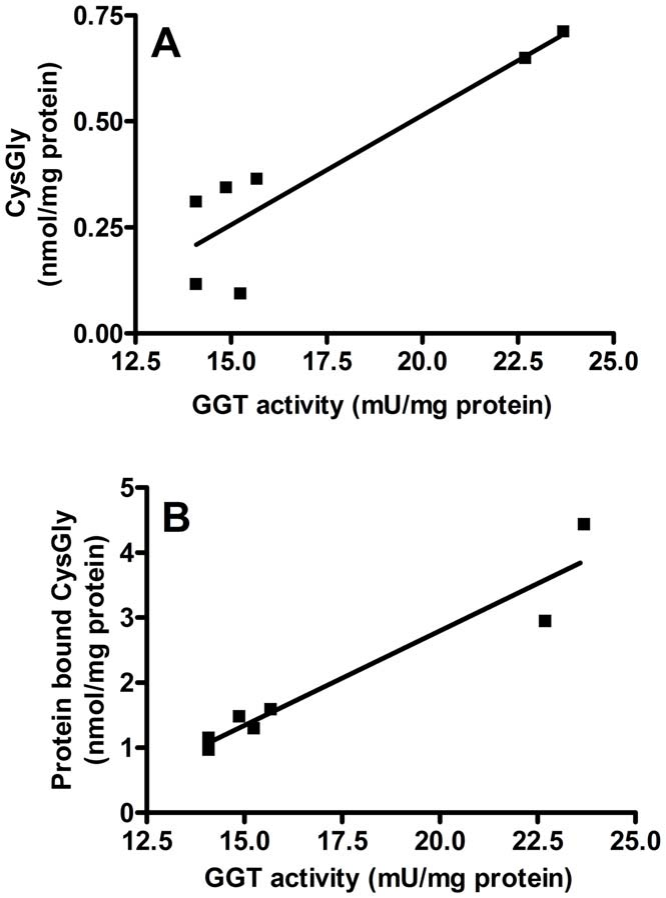
Relationship between GGT activity and cysteinyl-glycine (CysGly) levels in whole sputum. Data were obtained from seven different samples of CF sputum. (A) Free and (B) protein bound CysGly. A) R^2^ = 0,811, p<0.01; B) R^2^ = 0,917, p<0.001.

**Figure 2:**
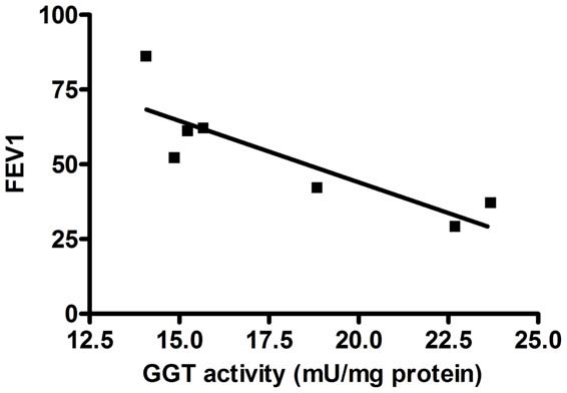
Relationship between sputum GGT activity and FEV1 values of CF patients. R^2^ = 0,717, p<0.02.

The analysis of the whole CF sputum homogenates revealed the presence of a mean GGT activity of 17.2±4.1 mU/mg of protein. The presence of a catalytically active GGT in CF sputum was also confirmed by the significant correlation between GGT activity and both free cysteinyl-glycine (R^2^ = 0.811, p<0.01; **Fig. 1A**) and protein bound cysteinyl-glycine (R^2^ = 0.917, p<0.001; **Fig. 1B**), the latter being about five times higher than the free compound. Interestingly, a significant (R^2^ = 0.717, p<0.02), inverse correlation was found between sputum GGT and FEV1 values of enrolled patients (**Fig. 2**).

**Figure 3:**
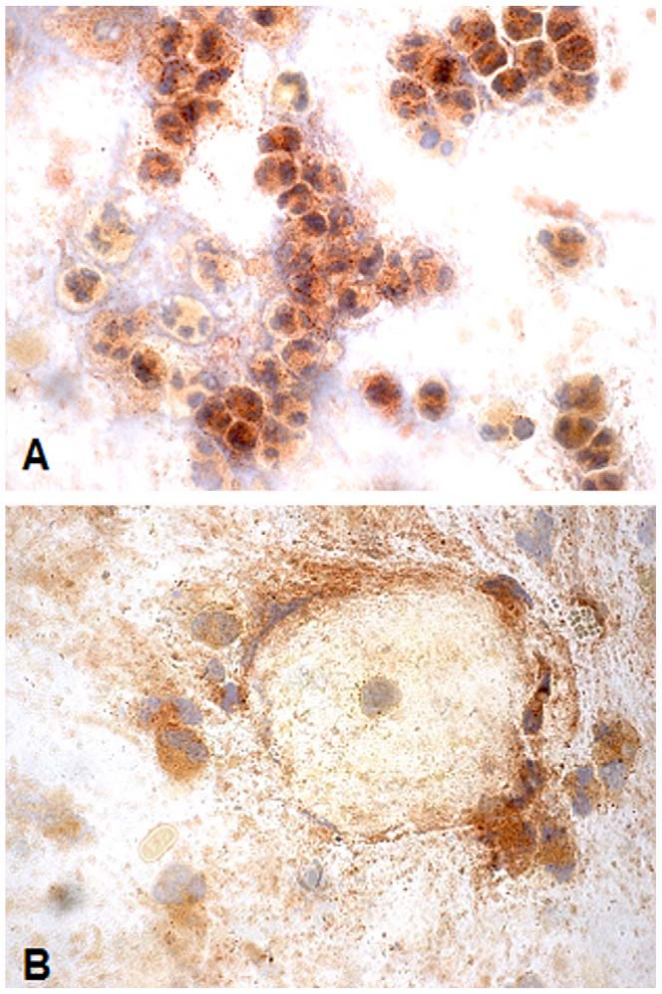
Cytochemical staining for GGT enzyme activity in sputum samples. (A) Neutrophils with different levels of GGT activity in sputum films of patients with cystic fibrosis. (B) GGT-negative epithelial cell surrounded by GGT-positive neutrophils is also shown. Magnification 100×.

As expected, sputum smears revealed the presence of bacteria, epithelial cells and a rich neutrophilic infiltrate, the latter expressing significant levels of GGT activity (**Fig. 3**). No correlation was found between GGT activity and microbiological parameters (type of microorganism, early or chronic infection; see **Table 1**).

**Table 1:** Microbiological characterization of CF sputum samples.

Pt#	Pseudomonas aeruginosa	Staphylococcus aureus
1	No	Yes, chronic
2	Yes, chronic	Yes, chronic
3	No	No
4	Yes, chronic	Yes, chronic
5	Yes, chronic	No
6	Yes, chronic	No
7	Yes, chronic	No

### Characterization of cell-free GGT activity in CF sputum

**Figure 4:**
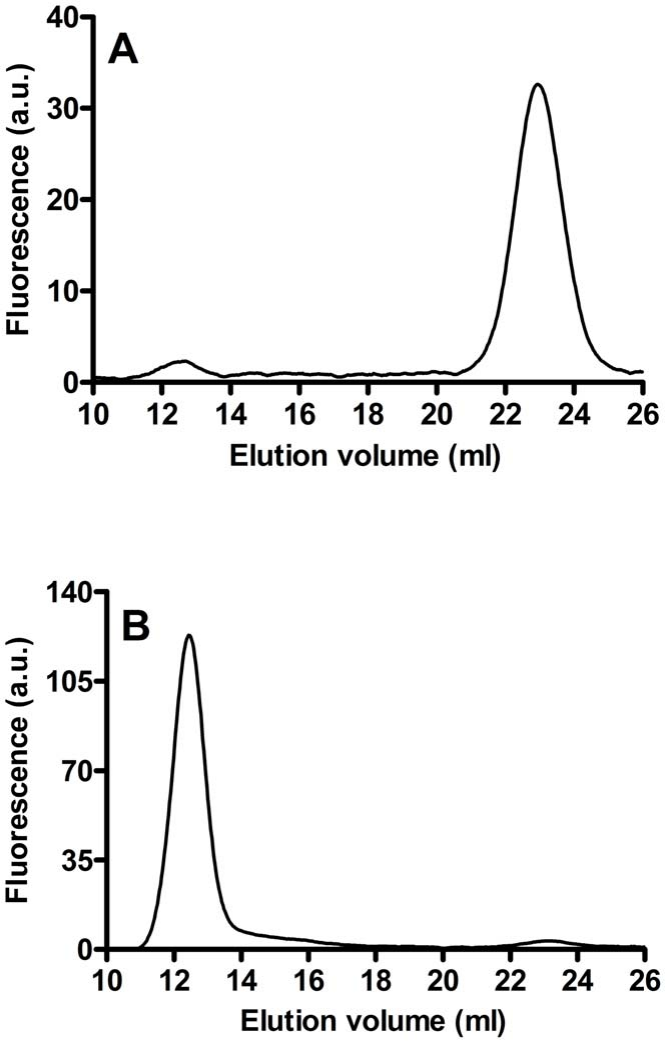
High-performance gel filtration chromatography of soluble fraction of CF sputum. Supernatants obtained from sputum solubilisation and centrifugation at 10,000×g were ultracentrifuged again at 100,000×g before analysis. A) 100,000×g supernatant; B) 100,000×g pellet. Data represent one representative separation out of three and are expressed as arbitrary units (a.u.) of fluorescence.

**Figure 5:**
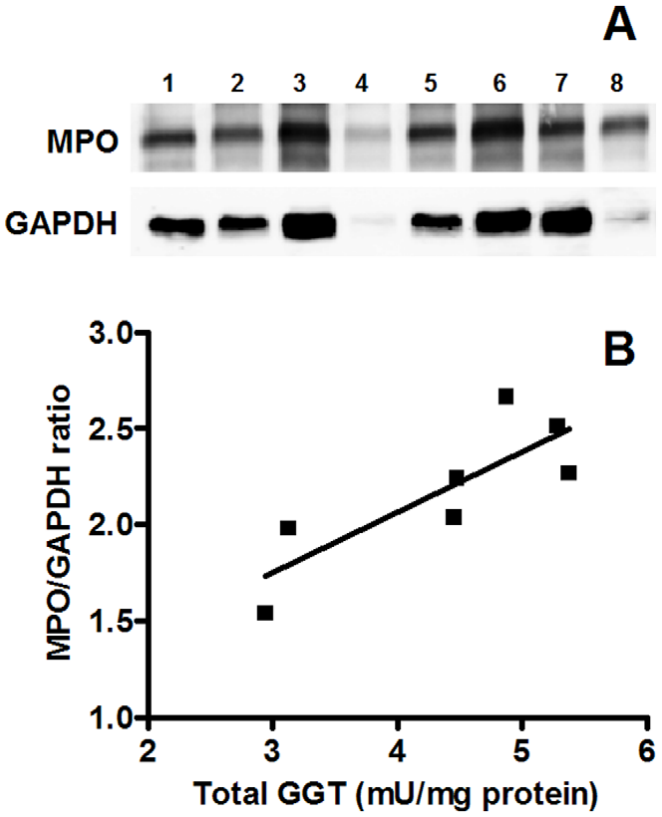
Relationship between GGT activity and MPO levels in CF sputum samples. MPO levels were detected by western blot analysis in samples of solubilised sputum pellets and correlated with GGT activity of solubilised sputum supernatants. A) Lane 1–7, CF samples; lane 8, control (neutrophils). B) Data reported are expressed as a *ratio* of MPO against GADPH band densities, while GGT values are normalized on protein content. R^2^ = 0.683; p = 0.02.

Gel-filtration chromatography of solubilised, cell-free sputum samples revealed the presence two peaks of GGT activity eluting respectively at 12.5 ml (“b-GGT”, MW>2000 kDa) and at 23.1 ml (“f-GGT”, 66 kDa) (**Table 2**). The same two peaks were also found in bronchiectasis sputum samples used as control (data not shown). The ratio between the two fractions varied considerably among the samples analyzed, b-GGT being anyway the prevalent fraction (**Table 2**). Gel-filtration chromatography of ultracentrifuged solubilised sputum showed that b-GGT fraction was mainly (90%) recovered in the pellet (**Fig. 4A–B**), while f-GGT was almost totally found in the supernatant (**Fig. 4A**). Interestingly, when MPO expression in cellular fraction of solubilised sputum were analyzed by SDS-PAGE, a significant correlation (R^2^ = 0.683; p = 0.02) was found with total GGT activity in the supernatants (**Fig. 5**). A significant correlation (R^2^ = 0.594; p = 0.04) was also found between MPO levels and GGT activities revealed in solubilised sputum supernatants (data not shown).

**Table 2:** Total and fractional GGT activity in CF sputum.

Pt#	Total GGT	b-GGT	f-GGT
1	13.1	5.2	4.7
2	19.6	9.8	5.2
3	21.6	14.1	2.1
4	28.1	15.8	12.2
5	48.2	29.3	18.9
6	84.1	46.8	37.3
7	90.2	50.9	30.4

CF sputum samples were solubilised, centrifuged at 10,000×g and the supernatants analyzed by high-performance gel filtration chromatography. The table reports the whole GGT activity of each solubilised sputum and the activities corresponding to the two different GGT fractions identified by gel filtration chromatography. Data were are expressed as U/L.

### Characterization of GGT activity in resting and activated neutrophils

**Figure 6:**
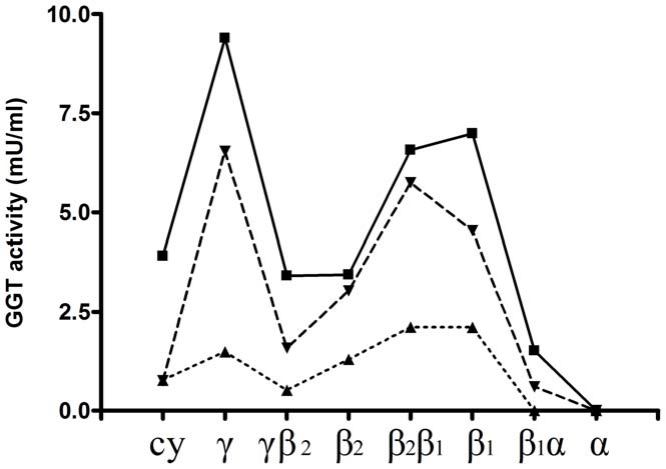
GGT activity in neutrophils fractions obtained on Percoll gradients. A high GGT activity was found in fractions γ, β_1_ and in the fractions between β_2_ and β_1_ (β_2_β_1_). Data reported were obtained from neutrophils isolated from three different healthy donors. Cy = cytosol; γ = secretory vesicles and plasma membranes; β_2_ = gelatinase granules; β_1_ = specific granules; α = azurophilic granules; γβ_2_, β_2_β_1_ and β_1_α are the fractions recovered among the main bands.

When a subcellular fractionation of neutrophils on a Percoll density gradient was performed, the presence of GGT activity was detected in the γ-band, containing secretory vesicles and plasma membranes, and in the β_1_-band, containing the specific granules (**Fig. 6**). Very low or no detectable GGT activity was found in α-band and β_2_-band, corresponding to azurophil and gelatinase granules, respectively.

**Figure 7:**
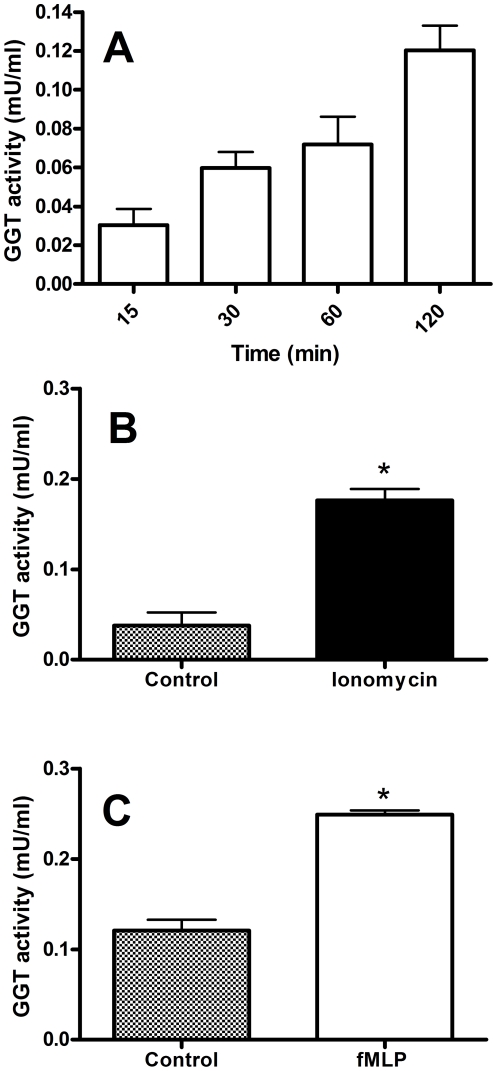
GGT release by neutrophils. Neutrophils isolated from fresh buffy coats were incubated in (A) RPMI-1640 alone, (B) in the presence of ionomycin (0.5 µM; 15 min) or (C) fMLP (1 µM; 120 min). GGT activity was measured in the 10,000×g centrifuged supernatants. Results are means ± SD of three separate determinations. Data were analyzed by Student's t test; (*) p<0.0001.

Neutrophils were then exposed to activating substances promoting granules release, and GGT activity was measured in the incubation media. A time-dependent release of GGT was observed in basal conditions (**Fig. 7A**), possibly as the result of a weak activation during incubations [17], [22]. Noteworthy, this effect was significantly increased when neutrophils were activated with the calcium ionophore ionomycin (**Fig. 7B**) or with the formyl peptide fMLP (**Fig. 7C**).

### Characterization of GGT released by activated neutrophils

**Figure 8:**
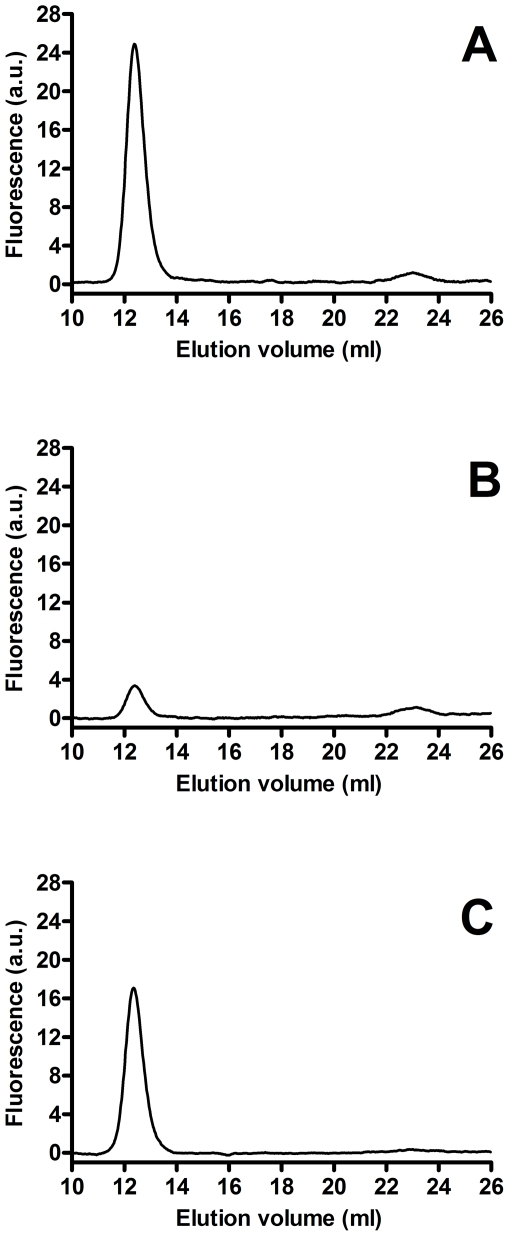
High-performance gel filtration chromatography of ionomycin activated neutrophils supernatants. Samples were centrifuged at 10,000×g or 100,000×g before analysis. (A) 10,000×g supernatant; (B) 100,000×g supernatant; (C) 100,000×g pellet.

In order to better characterize the GGT released by activated neutrophils, incubation media were centrifuged at 10,000×g, then at 100,000×g. The 10,000×g supernatants of both ionomycin (**Fig. 8A**) and fMLP (data not shown) activated neutrophils displayed the presence of one major peak of activity, corresponding to b-GGT observed in CF sputum, while only traces of f-GGT were detectable. On the contrary, the corresponding pellet showed no GGT activity (data not shown). When 100,000×g supernatants and pellets were analyzed, b-GGT was found in both fractions with a *ratio* of peak areas (corrected for the volumes) of 1∶1 (**Fig. 8B–C**). Again, only minor f-GGT peaks were found in the supernatants, while no f-GGT was detectable in the pellet (**Fig. 8B–C**).

### Comparison of sputum GGT with neutrophilic and epithelial GGT

**Figure 9:**
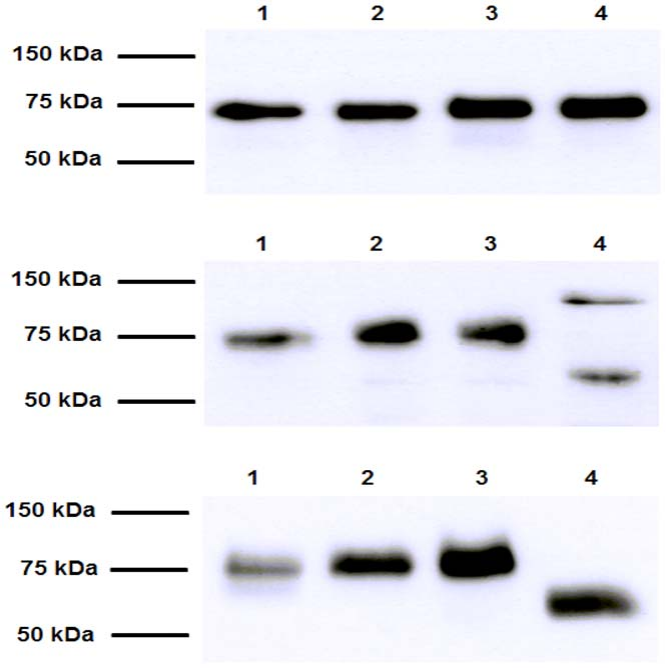
Western blot analysis of GGT heavy chain in different biological samples. (A) Lane 1–4, CF sputum; (B) lane 1, CF sputum; lane 2–3, neutrophils; lane 4, IB3-1 cells; (C) lane 1, soluble fraction of CF sputum; lane 2, insoluble fraction of CF sputum; lane 3, neutrophils; lane 4, A549 cells.

The possible origin of CF sputum GGT was investigated by means of SDS-PAGE analysis with an antibody directed against GGT heavy chain. Different whole sputum samples presented with a single band, corresponding to the MW of GGT heavy chain (75 kDa; **Fig. 9A**). A band with the same MW was observed in healthy donors neutrophils homogenates (**Fig. 9B**), in soluble/insoluble fractions of CF sputum (**Fig. 9C**) and in bronchiectasis sputum samples (data not shown). Conversely, a different MW was determined for GGT heavy chain in homogenates of different epithelial and endothelial cell lines used for comparison: CF bronchial epithelium IB3-1 cell line (**Fig. 9B**), human carcinoma epithelial cell line A549 (**Fig. 9C**) and human endothelial cell line HUVEC (data not shown), the latter employed as a model for the highly represented endothelial cells in the lung.

## Discussion

GGT plays an important role in the metabolism of GSH, S-nitrosoglutathione [10] and leukotrienes [9], i.e. compounds playing a central role as inflammatory mediators, and changes occurring in the compartmentation of this enzyme activity can therefore represent a critical process during the immune response. The results obtained in this study suggest that GGT activity present in CF sputum can originate – at least in part – from polymorphonuclear granulocytes, as a result of their accumulation and activation in CF airways. Increasing GGT levels in sputum were correlated with both free and protein-bound levels of cysteinyl-glycine (**Fig. 1A–B**), i.e. the highly reactive GSH catabolite produced by GGT in the extracellular compartment. The finding is in support of a direct role of GGT activity in modulating both low molecular weight thiols balance (**Fig. 1A**) and proteins thiols redox status (**Fig. 1B**) in CF lung. Previous studies have shown that GGT can produce the cysteinyl-glycylation of proteins, thus altering the levels of protein-bound GSH and the overall protein S-thiolation status [12]. In the case of sputum, such effects could play a role in modulating the function/solubility of airways proteins, such as thiols rich lung fluid mucins [30].

Notably, we found a significant, inverse correlation between sputum GGT activities and FEV1 values of corresponding patients (**Fig. 2**). Nevertheless, no correlation was found between GGT activity and parameters of microbial infection (see **Table 1**). The number of samples studied is quite small and future studies – enrolling a larger number of patients – will probably help to clarify these specific points. Anyway, functional data seems to associate the worsening of respiratory function with an increase of airways GGT, thus prompting the question of the source of sputum GGT (parenchimal or inflammatory).

In this respect, cytochemical staining for GGT activity confirmed the presence of rich GGT-positive neutrophilic infiltrates in all sputum samples. Neutrophils displayed different levels of the enzyme (**Fig. 3**), possibly ensuing from differences in GGT expression or activation. When solubilised cell-free samples were analyzed by gel-filtration chromatography (**Table 2**), two peaks of GGT activity were apparent displaying the same molecular weights of two of the four GGT fractions found in human plasma, b-GGT (MW>2000 kDa) and f-GGT (66 kDa) [24], the former possibly representing a high molecular weight protein aggregate and the latter corresponding to the free enzyme. The same two peaks were also observed in solubilised, cell-free samples of bronchiectasis patients sputum, used as a control for a neutrophils-dominated, chronic airway inflammation process (data not shown), thus suggesting an inflammatory origin of the observed findings, rather than a specificity for cystic fibrosis.

According with this interpretation, we found a significant correlation (R^2^ = 0.683; p = 0.02) between MPO expression in cellular fraction of solubilised sputum and total GGT activity in the supernatants (**Fig. 5**). MPO is a major constituent of neutrophil cytoplasmic granules and its activity is proposed to be a direct measure of neutrophil presence and an indirect indicator of lung injury [31]. In this perspective, our results only suggest a direct relationship between neutrophilic infiltrate and soluble GGT fractions in sputum. With the aim to ascertain whether neutrophils might be the source of that GGT, additional experiments were performed with isolated neutrophils.

In agreement with early reports [9], [20], our data confirmed the presence of GGT in neutrophilic granules. In particular, GGT activity was found in the subcellular fraction corresponding to secretory vesicles and plasma membranes (γ-fraction), as well as in specific granules (**Fig. 6**). Actually, the similar density of plasma membranes and secretory vesicles precludes the complete separation of these two components of γ-fraction, and further studies are needed to fully elucidate this specific point. On the other hand, it was shown that secretory vesicles are almost completely mobilized from neutrophils challenged with fMLP [22], and that specific granules are mobilized by calcium ionophore A23187 [20]. In our experiments, stimulation of isolated neutrophils with fMLP produced a time-dependent release of GGT activity (**Fig. 7C**), and the same was observed after treatment with calcium ionophore ionomycin (**Fig. 7B**), suggesting that the enzyme may indeed be associated with both secretory vesicles and specific granules of neutrophils. Gel-filtration chromatography of such activated neutrophils supernatants revealed the presence of one major GGT fraction, i.e. b-GGT (**Fig. 8**), corresponding to the same high molecular weight fraction found in cell-free sputum samples (**Table 2**).

Based on this evidence, the possible neutrophilic origin of the GGT fractions detected in cell-free sputum samples was further investigated. When neutrophils or their supernatants were compared with whole or solubilised CF sputum by SDS-PAGE, GGT heavy chain presented with the same MW in all sample analyzed, and this MW was different from GGT of epithelial (CF bronchial epithelial cells IB3-1 and human alveolar A549 cell line; **Fig. 9**) or endothelial origin (human endothelial cell line HUVEC; data not shown) used for comparison. Again, similar results were also obtained from bronchiectasis sputum samples used as control (data not shown). These findings are of particular interest, for post translational glycosylation of GGT protein – and thus its MW – is tissue specific [32], which allows a first, rough assessment of GGT proteins expressed in different tissues. An amphipathic GGT is physiologically secreted by alveolar epithelial type 2 cells in association with lung surfactant [33], and inflammation-related oxidative stress and cytokines can both induce GGT expression and release by lung epithelial cells [34]. Our results actually suggest that cell-free GGT in CF sputum can have a neutrophilic rather than epithelial origin, even if it can not be excluded that other GGT expressing inflammatory cells – such as macrophages [17], [35] – might also contribute to the phenomenon. Nevertheless, the rich neutrophilic infiltrate, the significant correlation between GGT activity and MPO expression and the ability of activated neutrophils to release soluble GGT – with biochemical characteristics similar to sputum GGT – are all in support of a neutrophilic origin of GGT. This effect may be of particular relevance – even though not specifically related – in cystic fibrosis, where eosinophils as well as neutrophils have been suggested to have an increased propensity to release their granule proteins (ECP and MPO), due to still unknown priming mechanisms (e.g. cytokines stimulation or upregulation of CR3-receptors) [36].

**Figure 10:**
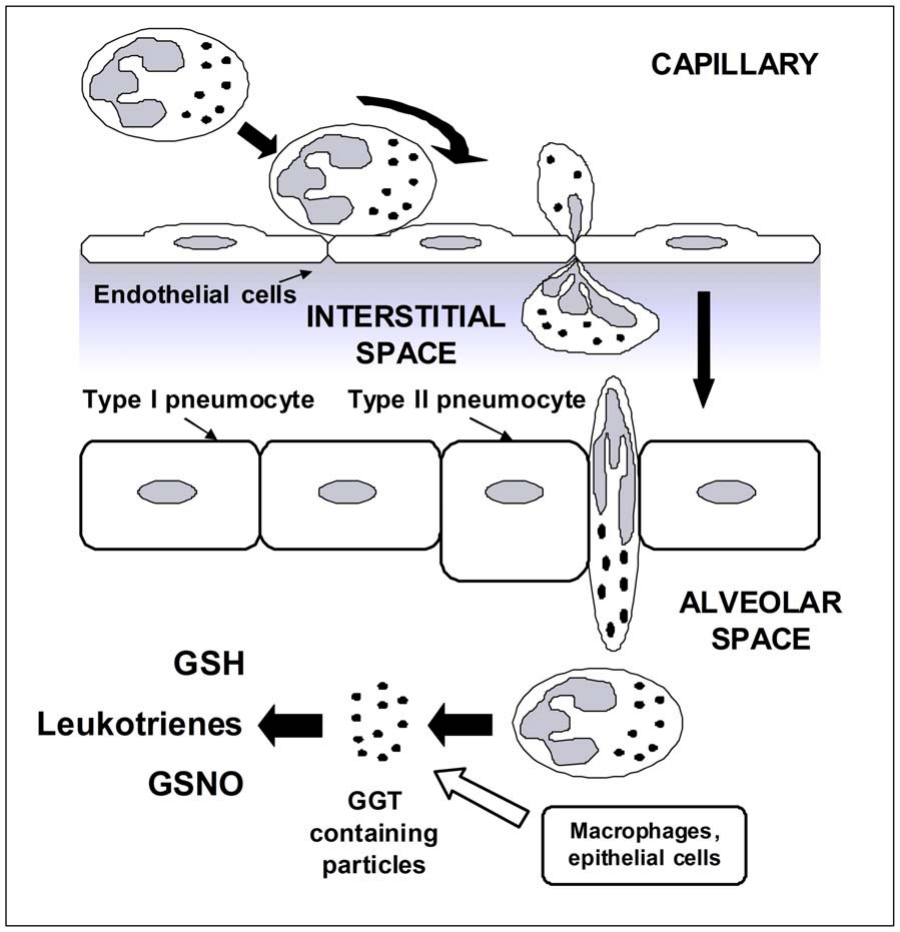
Neutrophils activation as a possible source of GGT in the airways during inflammation. *GSNO*, S-nitrosoglutathione.

As regards GGT fractions found in both activated neutrophils supernatants and cell-free sputum samples, most of b-GGT was recovered in ultracentrifugation pellets (**Figs. 4** **,** **8**). The fact that variable amounts of b-GGT were detected in supernatants suggest a heterogeneous composition of such fraction, possibly due to different origin/composition (e.g. secretory vesicles *vs.* specific granules) or subsequent modification in the inflammatory exudate (sputum). Previous studies showed that several cell types can shed small vesicles, and two main vesicle-discharge processes were identified leading to the release of distinct vesicle types: i) exocytosis of multivesicular bodies, with the ensuing release of exosomes, and ii) direct budding from plasma membrane of ectosomes, also termed microparticles [37]. Mixed vesicle populations were shown to be released upon activation by different cell types, and the presence of released vesicles has been detected in different body fluids such as urine, bronchoalveolar lavage fluid, saliva and blood [37]. Ectosomes were shown to be released by neutrophils [22], [38] and their involvement in different functions in the immune response was proposed [37]. This could indeed be also the case of neutrophilic GGT that – similarly to transmembrane receptor CR1 [22] – is comprised in complexes released upon cell activation with ionomycin or fMLP (**Fig. 7**). In this way GGT activity could be increased in the exudate more rapidly than in the case of its induction in parenchymal cells, which could help to early modulate inflammatory response through GGT substrates metabolism (**Fig. 10**).

The low mol. weight fraction f-GGT was recovered only from ultracentrifugation supernatants (**Figs. 4** **,** **8**). It can be envisaged that f-GGT might derive from the proteolytic cleavage of larger aggregate b-GGT by proteases released during immune response. In agreement with this interpretation, f-GGT was mainly found in CF sputum (**Fig. 4**), while only traces were detectable in short-term activated neutrophils supernatants (**Fig. 8**).

In conclusion, our data indicate that neutrophilic infiltrates can explain the increase of GGT activity in neutrophils-dominated airway inflammation processes, such those commonly observed in CF lungs. GGT is promptly released upon neutrophil activation, and this may have rapid consequences on all GGT substrates, including major inflammatory mediators. In this perspective, GGT increase in tissues should be interpreted not only as a consequence of inflammation related oxidative stress, but also as one of the effects of immune response. Depending on what effects the increase in this enzyme activity might produce on selected mediators, GGT could conceivably represent an interesting pharmacological target in order to modulate the inflammatory process. Further studies are however needed to fully elucidate the mechanisms of GGT release, the composition of GGT-containing particles and their actual role(s) in the inflammatory process.
